# High-Throughput Screening for the Prevalence of Neutralizing Antibodies against Human Adenovirus Serotype 5

**DOI:** 10.3390/vaccines12020155

**Published:** 2024-02-01

**Authors:** Jochen M. Wettengel, Hiroaki Naka, Gregory A. Dissen, Jeffrey Torgerson, Michelle Pounder, Simon F. Mueller, Elisabeth Mueller, Philipp Hagen, Micah Brandt, Ulrike Protzer, Benjamin J. Burwitz

**Affiliations:** 1 Division of Pathobiology and Immunology, Oregon National Primate Research Center, Oregon Health & Science University, Beaverton, OR 97006, USA; wettengel@tum.de (J.M.W.);; 2 Institute of Virology, Technical University of Munich, 81675 Munich, Germany; 3 German Center for Infection Research, Munich Partner Site, 81675 Munich, Germany; 4 Division of Genetics, Oregon National Primate Research Center, Oregon Health & Science University, Beaverton, OR 97006, USA; nakah@ohsu.edu; 5 Molecular Virology Core, Oregon National Primate Research Center, Oregon Health & Science University, Beaverton, OR 97006, USA; disseng@ohsu.edu (G.A.D.); torgersj@ohsu.edu (J.T.);; 6 LABOKLIN GmbH, 97688 Bad Kissingen, Germany

**Keywords:** adenoviral vector, adenoviral vaccine, adenoviral immunity, neutralizing antibodies

## Abstract

Adenoviral vectors based on the human adenovirus species C serotype 5 (HAdV-C5) are commonly used for vector-based gene therapies and vaccines. In the preclinical stages of development, their safety and efficacy are often validated in suitable animal models. However, pre-existing neutralizing antibodies may severely influence study outcomes. Here, we generated a new HAdV-C5-based reporter vector and established a high-throughput screening assay for the multivalent detection of HAdV-C5-neutralizing antibodies in serum. We screened the sera of rhesus macaques at different primate centers, and of rabbits, horses, cats, and dogs, showing that HAdV-C5-neutralizing antibodies can be found in all species, albeit at different frequencies. Our results emphasize the need to prescreen model animals in HAdV-C5-based studies.

## 1. Introduction

Viral vectors have been integral to clinical applications such as gene or cancer therapy and vaccination [1]. While several viral vectors are used in vaccine development, adenoviral vectors are among the most studied [2,3,4,5,6,7,8].

Adenoviruses are non-enveloped, linear, double-stranded DNA viruses with large genome sizes between 26 and 45 kb [9]. Compared to other vector-based vaccines, adenoviruses offer several advantages. First, numerous established methods exist to quickly construct, generate, purify, and titrate adenoviral vectors [10,11,12]. Second, adenoviral vectors exhibit relatively low pathogenicity, limited integration potential, and strong immunogenicity [13]. Finally, adenoviral vectors show a high genetic stability [14,15] and can carry large transgenes. These advantages have made adenoviruses a staple in vaccine research, including recent efforts to design vector-based vaccines against SARS-CoV-2 [16].

The broad application of adenoviral vectors has led to improvements in packaging capacity. First-generation adenoviral vectors with deletions of the E1 and E3 regions can carry up to 8.2 kb of foreign DNA [15]. Further deletions in the E2 and E4 regions led to second-generation adenoviral vectors [17], which exhibit an increased packaging capacity of up to 14 kb [18]. While first- and second-generation adenoviral vectors still express adenoviral genes and could lead to unwanted immune responses, third-generation or “gutless” adenoviral vectors no longer express endogenous adenoviral genes and have a transgene capacity of up to 37 kb [19,20].

Adenoviral-based therapies and vaccines must be designed around the numerous available adenovirus species and serotypes from different animal species, each with specific host and tissue tropisms. The most characterized adenovirus is the human adenovirus species C serotype 5 (HAdV-C5), which was recently used to develop the recombinant viral vectors for the SARS-CoV-2 vaccine candidates, like the approved vaccines Convidecia and Sputnik V in China and Russia, respectively [21]. Clinical studies showed that the prevalence of pre-existing high-titer HAdV-C5-neutralizing antibodies could compromise the seroconversion of SARS-CoV-2-neutralizing antibodies post-vaccination [22,23]. HAdV-C5 was also used as the viral vector in the HIV STEP trial [24], which was ended when predetermined non-efficacy endpoints were met. A post-hoc analysis showed that patients with prior HAdV-C5-neutralizing antibodies acquired HIV more readily than the comparable placebo group [25].

These findings highlight the need to understand the interplay between adenovirus neutralization and adenoviral-based therapies and imply that adenoviral immune responses may significantly affect the therapy outcome.

Commercially available enzyme-linked immunosorbent assays (ELISAs), chemiluminescence immunoassays (CLIAs), and chemiluminescent microparticle immunoassays (CMIAs) have been optimized to offer standardized and fast methods to detect antibodies against adenoviruses in serum. These assays are based on single or multiple antibody/antigen interactions but cannot measure the neutralization potential of antibodies. Moreover, many of these assays are not serotype-specific, and cross-reactive antibodies might lead to false-positive results. Therefore, cell-culture-based assays are the gold standard to detect neutralizing antibodies against a specific serotype.

While several HAdV-C5 neutralization assays have been developed in the past, they usually use single reporter readouts, have limited dynamic ranges, or are unsuitable for high-throughput studies [26,27,28,29]. Additionally, most of these assays are labor-intensive, expensive, and time-consuming, leading to variable results. Thus, there is a critical need for a reliable, multi-valent, high-throughput screening approach that identifies the prevalence of neutralizing antibodies against adenoviral vectors, helping to select suitable animals or patients for preclinical or clinical testing with HAdV-C5-vectors, respectively.

Here, we established a high-throughput screening assay for the multivalent detection of HAdV-C5-neutralizing antibodies in serum via a moxGFP fluorescent protein and a NanoLuc luciferase. We screened rhesus macaques from the Oregon National Primate Research Center (ONPRC) and other US national primate centers for the prevalence of neutralizing antibodies against HAdV-C5 and extended this screening to other species. We found that the prevalence of pre-existing neutralizing HAdV-C5-antibodies varies across species and, in the case of rhesus macaques, depends on the primate center of origin.

## 2. Materials and Methods

### 2.1. Generation of the HAdV-C5-Reporter Virus

Synthesized DNA fragments consisting of a transthyretin (TTR) promotor, a moxGFP gene with a nuclear localization signal (NLS), a P2A-site, and a NanoLuc luciferase gene were cloned into a pENTR4 vector (Thermo Fisher Scientific, Waltham, MA, USA, A10465). The Gateway LR Clonase II Enzyme Mix (Invitrogen, Waltham, MA, USA, 11791100), in combination with the pAd/PL-DEST Gateway Vector Kit (Invitrogen, V49420), was used to generate the pAd-TTR-moxGFP-P2A-NanoLuc plasmid, encoding a replication-deficient recombinant first-generation adenovirus serotype 5 (ΔE1/ΔE3).

Adenoviral crude stock was generated by transfecting HEK293A cells (Life Technologies, Carlsbad, CA, USA, R70507), plated on a 12-well plate (Falcon, Arlington, VA, USA, 353043), using Lipofectamine 3000 (Thermo Fisher Scientific, L3000015) with the PacI-digested (Thermo Fisher Scientific, FD2204) pAd-TTR-moxGFP-P2A-NanoLuc plasmid according to the supplier’s protocol. On the next day, cells were transferred to a T25 flask and further incubated. Approximately 5–8 days post-transfection, cytopathic effects were visible in the cell layer, and the flask was further incubated for 3–4 days until most of the cells detached. The cell suspension was harvested and frozen/thawed three times, resulting in approximately 5 mL adenoviral crude stock.

For further amplification, HEK293A cells were plated on a T125 flask and inoculated with 2 mL adenoviral crude stock. After 2–3 days, the cell suspension was collected and centrifuged. The supernatant was discarded, and the cell pellet was resuspended in 500 µL PBS. The virions were harvested from the cells by taking the cells through three freeze/thaw cycles. To receive an adenovirus working stock, the sample was centrifuged, and the supernatant was aliquoted.

To achieve higher adenovirus concentrations, adenovirus working stocks were combined and treated with benzonase nuclease (Millipore, Burlington, MA, USA, E1014-5KU) to digest genomic DNA. The virions were purified by ultracentrifugation over two cesium chloride gradients as previously described [30,31]. The virions harvested from the second gradient were dialyzed against a physiological buffer, 10 mM tris pH 8.0 (Fisher, BP2473-100), 2 mM magnesium chloride (Sigma Aldrich, St. Louis, MO, USA, 7786-30-3), and 4% sucrose (*w*/*v*) (EMD, Darmstadt, Germany, SX1075-1) [30].

### 2.2. Titration of the HAdV-C5-Reporter Virus Stock

The titers of infectious HAdV-C5-reporter virions were determined using a half-maximal tissue culture infectious dose (TCID50) and plaque assays.

For the TCID50 assay, HEK293 cells were seeded on 96-well plates in 100 µL DMEM/F-12 (Gibco, New York, NY, USA, 11320-033) supplemented with 10% heat-inactivated fetal bovine serum (FBS) (Neuromics, Edina, MN, USA, FBS002), 1% Antibiotic/Antimycotic Solution (Cytiva, Marlborough, MA, USA, SV30079.01), and 1% MEM Non-Essential Amino Acids Solution (Cytiva, SH30238.01) and incubated for 24 h at 37 °C and 5% CO_2_. On the next day, the cells were inoculated with a 10-fold dilution series of the virus stock in eight wells each and further incubated for 1–2 weeks. The luciferase expression in each well was quantified (see Section 2.7) and compared to visible cytopathic effects (CPEs). TCID50 titers were calculated using the Spearman–Karber method as previously described [32].

For the plaque assay, HEK293 cells were seeded on six-well plates in 2 mL DMEM/F-12 (Gibco, 11320-033) supplemented with 10% heat-inactivated fetal bovine serum (FBS) (Neuromics, FBS002), 1% Antibiotic/Antimycotic Solution (Cytiva, SV30079.01), and 1% MEM Non-Essential Amino Acids Solution (Cytiva, SH30238.01) and incubated for 24 h at 37 °C and 5% CO_2_. The cells were inoculated with a 10-fold dilution series of the virus stock the next day and further incubated for 24 h. Next, the media were exchanged with 2 mL DMEM/F-12 (Gibco, 11320-033) supplemented with 1% carboxymethylcellulose and 1% Antibiotic/Antimycotic Solution (Cytiva, SV30079.01) and incubated for 1–2 weeks at 37 °C and 5% CO_2_. After visible CPEs, cells were fixed and stained with a 1% crystal violet (*w*/*v*) solution to count the plaques per well.

### 2.3. Serum Samples

Serum samples from macaques were leftover routine diagnostic samples obtained by the National Primate Research Centers from Oregon (ONPRC), Wisconsin (WNPRC), Emory (ENPRC), and Tulane (TNPRC). Serum samples from rabbits, horses, dogs, and cats were leftover routine diagnostic samples obtained by LABOKLIN GmbH. Serum samples from humans were leftover routine diagnostic samples obtained by the Institute of Virology at the Technical University of Munich. Human samples were anonymized prior to use. To inactivate complement proteins, all sera were heat-inactivated for 30 min at 56 °C.

### 2.4. HAdV-C5-Neutralization Assay

For the HAdV-C5-neutralization assay (Figure S1), HepG2 cells (ATCC, Manassas, VA, USA, HB-8065) were seeded at 80% confluence (approx. 3 × 10^4^ cells/well) onto flat-bottom 96-well plates (Falcon, 353072) pretreated with a collagen I solution (Gibco, A10483-01) diluted to 0.3 mg/mL in UltraPure DNase/RNase-Free Distilled Water (Invitrogen, 10977049). Cells were cultured in 100 µL DMEM/F-12 (Gibco, 11320-033) supplemented with 10% heat-inactivated fetal bovine serum (FBS) (Neuromics, FBS002), 1% Antibiotic/Antimycotic Solution (Cytiva, SV30079.01), and 1% MEM Non-Essential Amino Acids Solution (Cytiva, SH30238.01) overnight at 37 °C.

The next day, the HAdV-C5-based reporter vector was diluted in cell culture media to obtain a suitable concentration (we recommend a multiplicity of infection (MOI) of one infectious particle/cell). This mixture was added to the serum samples in 96-well plates or sample tubes in a proper ratio (depending on the experimental set-up, we recommend starting with a 20% serum concentration) and incubated at room temperature for 1 h with agitation. In the last step, 25 µL of the mixture was added to the 100 µL of media in the 96-well HepG2 plates (to obtain a total of 4% serum concentration), which were subsequently incubated at 37 °C for 48–96 h (we recommend 48 h). The assay was analyzed through fluorescence microscopy, flow cytometry, or NanoLuc luminescence analysis.

### 2.5. Fluorescence Microscopy

MoxGFP-NLS expression and nuclear localization were analyzed via fluorescence microscopy on a Leica DM8i (Leica Biosystems, Buffalo Grove, IL, USA, DM8i). Fluorescence images were obtained using the Leica Application Suite X (Leica Biosystems, LAS X).

### 2.6. Flow Cytometry

Following the 48–96-h incubation with the HAdV-C5-based reporter vector, HepG2 cells were trypsinized and stained with a LIVE/DEAD Viability/Cytotoxicity Kit (Invitrogen, L3224). Live cells were then collected on a Becton-Dickinson LSRII, and MoxGFP-NLS expression was analyzed using the FlowJo X software (Becton-Dickinson, Ashland, OR, USA).

### 2.7. NanoLuc Luminescence Analysis

A luminescence-based assay was used to analyze luciferase expression in relative light units (RLUs). For this assay, 10 μL cell culture supernatant was added on a white 96-well plate and mixed with 100 μL PBS-T (0.1% Tween-20) containing 1 μM Coelenterazine H stock solution (PJK Biotech, Kleinblittersdorf, Germany) dissolved in acidified methanol as previously described [33]. Luminescence was detected subsequently using a GloMax Navigator Microplate Luminometer (Promega, Madison, WI, USA, GM2010) or a Tecan Infinite 200 pro reader (Tecan Group, Maennedorf, Switzerland, 30050303).

### 2.8. Cell-Titer Blue Cell Viability Assay

Cell viability was measured using the Cell Titer-Blue Cell Viability Assay (Promega, G8080). The assay was performed according to the manufacturer’s instructions. The read-out was performed on a Tecan Infinite 200 pro reader (Tecan Group, 30050303), measuring fluorescence at 560/590 nm, or on a Synergy HTX, measuring absorbance at 573/602 nm.

### 2.9. Anti-HAdV-C5 IgG ELISA

For the anti-HAdV-C5 IgG ELISA, 96-well plates were incubated with 50 µL of coating buffer (30 mM Na_2_CO_3_, 70 mM NaHCO_3_, in H_2_O pH 9.5) mixed with 5% of the adenovirus working stock at 4 °C overnight. On the next day, the plate was washed three times with 2% BSA in PBS and blocked with 5% goat serum in PBS for 2 h at room temperature. Patient sera were added to the wells in a 1:25 dilution in 5% goat serum in PBS and incubated for 4 h at 4 °C. Subsequently, cells were washed five times with 2% BSA in PBS and incubated with a goat anti-human IgG-HRP (Santa Cruz Biotechnology, Dallas, TX, USA, sc-2453) for 1 h at 4 °C. In the last step, the wells were washed again five times with 2% BSA in PBS, developed with 100 µL of TMB substrate (Thermo Fisher Scientific, N301), and stopped with 50 µL 2 M sulfuric acid.

## 3. Results

**Figure 1:**
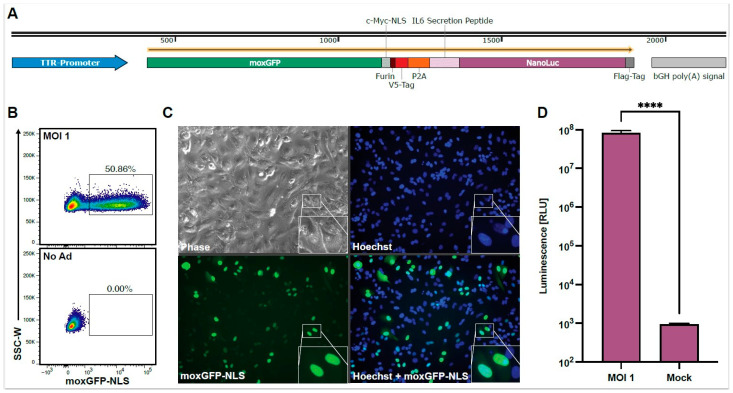
Overview of the HAdV-C5-based reporter vector and its multiple readout options. HepG2 cells were transduced with the HAdV-C5-base reporter vector at an MOI of one infectious particle/cell, and the expression of the reporter proteins was analyzed 96 h post-transduction. (**A**) The expression cassette of the HAdV-C5-based reporter vector. A murine transthyretin (TTR) promoter drives moxGFP, which is fused to a c-Myc nuclear localization signal (NLS) and connected to a NanoLuc luciferase, which is fused to an N-terminal IL-6 secretion signal. Both reporter proteins are connected via a linker region containing a Furin cleave site, a V5-Tag, and a P2A site. (**B**) moxGFP expression and the percentage of moxGFP-positive cells can be analyzed via flow cytometry. (**C**) moxGFP expression and moxGFP-positive cells can also be monitored via fluorescence microscopy. Through the fused NLS, moxGFP is localized in the nucleus, leading to an improved signal-to-noise ratio and allowing an easy quantification of transduced cells. (**D**) HAdV-C5-based reporter vector transduction (N = 12) can also be monitored via the NanoLuc expression level through luminescence analysis. Statistical analysis was performed using the *t*-test. **** *p* < 0.0001.

To establish a high-throughput screening for neutralizing antibodies against HAdV-C5, we designed a replication-deficient first-generation HAdV-C5-based reporter vector expressing different reporter proteins for multiple readouts. The reporter cassette (Figure 1A) is driven by the transthyretin (TTR) promoter, leading to robust gene expression in HepG2 cells, which are known for their high HAdV-C5-susceptibility [34]. We chose the fluorescent protein moxGFP as our first reporter protein due to its intensity, rapid maturation time, and monomeric protein structure, which is preferred for flow cytometric readouts (Figure 1B) [35]. Furthermore, we fused moxGFP to a c-Myc nuclear localization signal (NLS) [36] for a focused nuclear localization, allowing for the easy quantification of transduced cells by fluorescence microscopy (Figure 1C). We chose NanoLuc luciferase as our second reporter protein, which includes an N-terminal IL-6 secretion signal, offering a highly sensitive and cost-efficient readout from culture supernatant at multiple time points (Figure 1D) [37]. We connected both reporter proteins via a linker region containing a Furin cleavage site, a V5-Tag, and a P2A site for simultaneous and equimolar protein expression and analysis as previously described [38].

### 3.1. Optimization of Assay Parameters

**Figure 2:**
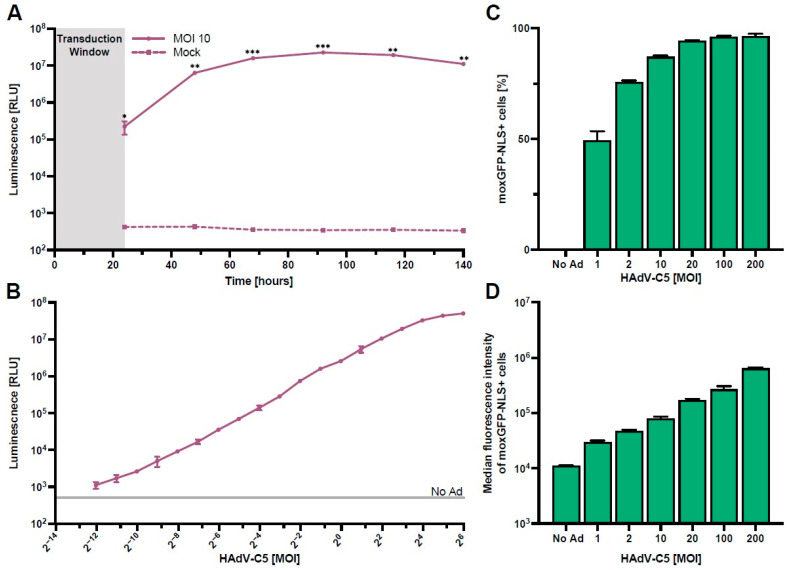
Optimization of assay parameters. HepG2 cells were transduced with the HAdV-C5-based reporter vector (N = 3). (**A**) NanoLuc expression kinetics after transduction with an MOI of one infectious particle/cell for 24 h. Supernatants were collected and exchanged every day, and NanoLuc luminescence was quantified. (**B**) NanoLuc luminescence quantified 48 h post-transduction using different vector concentrations. (**C**,**D**) Frequency and median fluorescence intensity of moxGFP-NLS expressing cells 96 h post-transduction using different vector concentrations. Statistical analysis was performed using the *t*-test, * *p* < 0.05, ** *p* < 0.01, and *** *p* < 0.001.

Using our HAdV-C5-based reporter vector, we next optimized the assay parameters required for rapid and high-throughput screening of serum antibody neutralization. We first performed a kinetics experiment to determine the time of peak NanoLuc expression in HepG2 cells. Here, we found that maximum expression is at approximately 96 h post-transduction, while a practical signal-to-noise ratio was already achieved between 24 h and 48 h post-transduction (Figure 2A).

Following the measurements of NanoLuc expression kinetics, we next analyzed the magnitude of NanoLuc secretion at 48 h post-transduction using different MOIs (Figure 2B). Our results show a clear linear relationship in NanoLuc relative light units (RLUs) between 0.001 and 8 infectious particles/cell, indicating a high dynamic range of this read-out. Since high HAdV-C5-reporter virus titers lead to cytotoxicity (Figure S2), we recommend the use of an MOI of one infectious particle/cell for the assay.

To define the number of HAdV-C5-transduced HepG2 cells more thoroughly, we analyzed the frequency and median fluorescence intensity of moxGPF-NLS expressing cells at 96 h post-transduction with multiple MOIs (Figure 2C,D).

In line with the NanoLuc data (Figure 2B), we found that the frequency of moxGFP-NLS+ cells increased asymptotically with higher MOIs, approaching 100% as the maximum, while the median fluorescence intensity of moxGFP-NLS+ cells increased gradually with escalating MOIs. This result indicates that assays utilizing moxGFP-NLS+ cell frequency as an endpoint should utilize lower MOIs, as higher MOIs lead to the saturation of target cells, and differences in neutralization are masked. However, the use of flow cytometry does allow for the use of higher MOIs since differences in median fluorescence intensity can still be inferred.

Directly comparing both methods for detecting HAdV-C5-reporter virus transduction in a high-throughput approach, we recommend using NanoLuc due to its high dynamic range and the option of readouts from culture supernatant at multiple time points.

### 3.2. Validation of HAdV-C5-Neutralization Measurements

**Figure 3:**
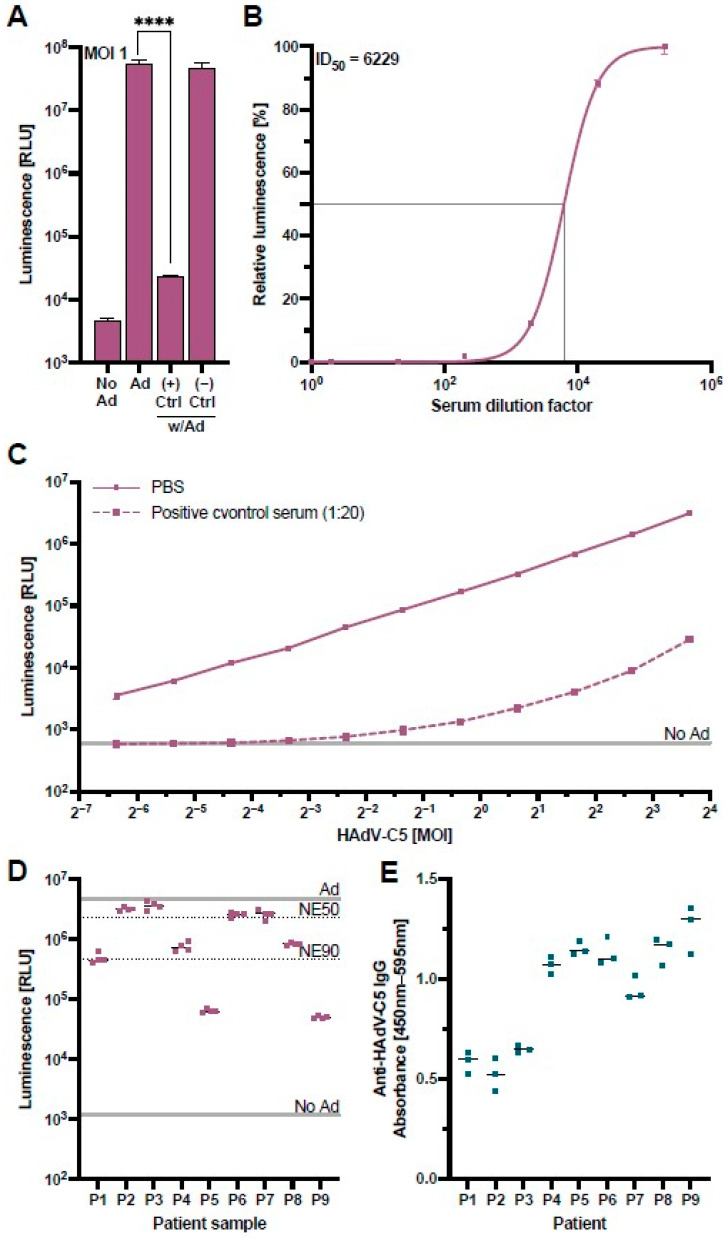
Validation of the HAdV-C5-neutralization assay. A control serum sample obtained from a monkey previously immunized with an HAdV-C5-based vector was used to validate the HAdV-C5-neutralization assay. (**A**) The HAdV-C5-based reporter vector was incubated at an MOI of one infectious particle/cell with control serum from a previously HAdV-exposed rhesus macaque (+) or a naïve rhesus macaque (−). HepG2 cells were transduced with the treated HAdV-C5-based reporter vector and the NanoLuc expression was analyzed at 96 h post-transduction (N = 8). Non-transduced cells (No Ad), transduction without serum (Ad), or incubation of HAdV-C5 with naïve rhesus macaque serum served as controls. Statistical analysis was performed using the *t*-test. **** *p* < 0.0001. (**B**) The positive control serum was serially diluted and incubated together with the HAdV-C5-based reporter vector at an MOI of one infectious particle/cell. HepG2 cells were transduced in triplicates, and the NanoLuc expression was analyzed at 96 h post-transduction. In addition, half-maximal inhibitory dilution (ID50) was calculated. (**C**) Different MOIs of the HAdV-C5-reporter vector were pre-incubated with 5% of the positive control serum and added to HepG2 cells (1% positive control serum on the cells) (N = 3). NanoLuc expression was analyzed at 48 h post-transduction. (**D**) Patient samples previously tested positive for pan-adenovirus-binding IgG were tested in the neutralization assay. The indicated lines represent controls of non-pretreated HAdV-C5-reporter vector (Ad), non-transduced cells (No Ad), 50% neutralization efficiency (NE50), and 90% neutralization efficiency (NE90). (**E**) Patient samples in panel D were tested for HAdV-C5-binding IgG through an ELISA.

To confirm that the assay could measure HAdV-C5-neutralization appropriately, we performed a validation experiment with control serum samples from a monkey previously immunized with an HAdV-C5-based vector and a naïve monkey with no known HAdV-C5 exposure [39]. The results of this validation confirmed that our HAdV-C5-based reporter vector co-incubated at an MOI of one infectious particle/cell with concentrations of 20% positive control serum during pre-incubation (4% positive control serum on the cells) led to a NanoLuc signal comparable to untransduced cells, while the negative control serum showed no suppression of the NanoLuc signal (Figure 3A). A cell viability assay excluded a possible cytotoxic effect of the positive control serum (Figure S3).

To assess the positive control serum’s neutralizing capacity, we performed a serial dilution of the serum and incubated these samples with the HAdV-C5-based reporter vector at a constant MOI of one infectious particle/cell (Figure 3B). We observed that the neutralization of the HAdV-C5 reporter vector exhibited a serum-dose dependency, allowing the determination of a half-maximal inhibitory dilution (ID_50_) of 1:6229 which equals a serum concentration of 0.016%.

Since serum dilutions of less than 1:200 neutralized the vector with an efficiency of greater than 98% (Figure 3B), we further analyzed the capacity of our positive control serum at a higher dilution of 1:20 (5%) to neutralize variable MOIs of the HAdV-C5-based reporter vector. While neutralization of the HAdV-C5-based reporter vector at this lower concentration was still greater than 98% at MOIs of one and fewer infectious particles per cell, higher MOIs led to an increase in the NanoLuc expression, indicating that the neutralizing capacity of the serum can be overcome by higher concentrations of the HAdV-C5-based reporter vector (Figure 3C). In a subsequent experiment, we validated our HAdV-C5 neutralization assay with sera from nine patients with clinically confirmed anti-adenovirus IgG antibodies tested by the Virotech pan-serotype Adenovirus IgG/IgM ELISA. We ordered these patient samples from the lowest (P1) to the highest anti-adenovirus IgG concentration (P9). Interestingly, our neutralization assay showed little correlation with the concentration of pan-adenovirus-specific antibodies, either due to the specificity of antibodies to a different adenoviral serotype or due to non-neutralizing HAdV-C5-binding antibodies (Figure 3D). We therefore performed an optimized anti-HAdV-C5-specific IgG ELISA as previously described [40] and found that the levels of HAdV-C5 neutralization did not significantly correlate to the levels of HAdV-C5-binding IgG (*p* = 0.125) (Figure 3E). This confirmed that the anti-HAdV-C5-specific IgG ELISA detects a broad range of HAdV-C5-binding IgG antibodies, while our cell-culture-based neutralization assay can specifically detect the subpopulation of neutralizing antibodies, highlighting the advantage of our assay.

These results show that our HAdV-C5-neutralization assay allows for a direct comparison of neutralizing sera by either sera dilution or, if appropriate, by increasing MOIs of the HAdV-C5-based reporter vector. In addition, unlike serological ELISAs designed to measure total anti-adenoviral IgG, our functional assay provides insight into the neutralization capacity of antibodies present in a sample.

### 3.3. Neutralizing Antibodies against HAdV-C5 in Non-Human Primates

Rhesus macaques play a critical role in the preclinical testing of HAdV-C5-based therapies and vaccines. However, there are no reliable data on the prevalence of neutralizing antibodies against HAdV-C5-based vectors in this species.

**Figure 4:**
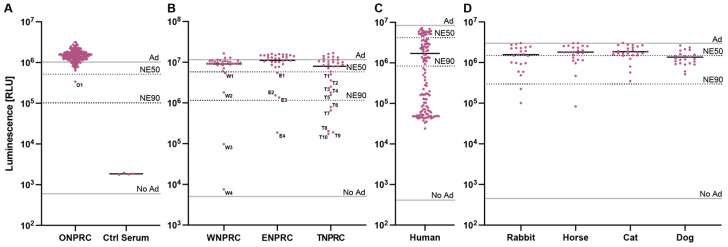
Screening of neutralizing antibodies against the HAdV-C5-based reporter vector. The established HAdV-C5 neutralization assay was performed with serum samples (**A**) drawn by routine examinations from rhesus macaques at the ONPRC colony, (**B**) received from different US-based national primate research centers, (**C**) from humans, and (**D**) from non-primate species. The indicated lines represent controls of non-pretreated HAdV-C5-reporter vector (Ad), non-transduced cells (No Ad), 50% neutralization efficiency (NE50), and 90% neutralization efficiency (NE90).

We therefore used our assay to determine the prevalence of HAdV-C5-neutralizing antibodies in sera of rhesus macaques. We first screened 178 serum samples drawn from different rhesus macaques at routine examinations at the Oregon National Primate Research Center (ONPRC) at a serum dilution of 1:5 (20%) with the HAdV-C5-based reporter vector at an MOI of one infectious particle/cell (Figure 4A). Out of the 178 screened samples, we identified a single serum sample (O1) (1/178—0.6%) neutralizing our HAdV-C5-based reporter vector with a >50% neutralization efficiency (NE50). However, the ID_50_ of O1 serum was 36, much lower than our positive control serum (Figure S4 and Figure 3B). This result indicates that most rhesus macaques in the ONPRC colony do not neutralize HAdV-C5.

We next performed a blinded screening of serum samples received from three other US-based national primate research centers located at the University of Wisconsin (WNPRC), Emory University (ENPRC), and Tulane University (TNPRC) (Figure 4B). Here, we identified several sera that neutralized our HAdV-C5-based reporter vector. We tested 32 serum samples from each primate center and saw >NE50 in 4 samples (W1-W4) (4/32—12.5%) from the WNPRC; in 4 samples (E1-E4) (4/32—12.5%) from ENPRC; and in 10 samples (T1–T10) (10/32—31.3%) from TNPRC. Sample unblinding revealed that the serum with the highest neutralization from all these samples (W4) came from a rhesus macaque previously administered an HAdV-C5-based vector, while the medical records of all other animals showed no HAdV-C5-based vector treatments. In summary, the overall prevalence of neutralizing sera (defined as >NE50) in our assay ranged from 0.6% to 31.3% across the different primate centers, and the prevalence of HAdV-C5-neutralizing antibodies in sera of all tested rhesus macaques was 7.3% (20/274). This result emphasizes that rhesus macaques should be screened for the prevalence of neutralizing antibodies against HAdV-C5 if used for studies including HAdV-C5-based vectors.

### 3.4. Neutralizing Antibodies against HAdV-C5 in Other Species

In a final experiment, we aimed to compare the prevalence of HAdV-C5-neutralizing antibodies in rhesus macaques to other species, including humans, rabbits, horses, cats, and dogs. As expected, we saw a high prevalence of pre-existing neutralizing antibodies (neutralization > 50%) in humans (99/120—82.5%), where HAdV-C5 infections are endemic (Figure 4C). However, we also detected pre-existing neutralizing antibodies in the sera of rabbits (11/23—47.8%), horses (7/23—30.4%), cats (5/26—19.2%), and dogs (16/23—69.6%), indicating that there is also a critical need to prescreen non-primate animals that are intended for use in HAdV-C5-based vector studies (Figure 4D).

## 4. Discussion

Adenoviral vectors have been developed as gene therapies and vaccines for various diseases, and several adenovirus-based treatments are currently under investigation in different preclinical phases. The most studied adenoviral vector is HAdV-C5 due to its ability to infect a broad range of different cell types and to generate potent vaccine responses. However, previous research in gene therapy and vaccinology has shown that pre-existing neutralizing antibodies to HAdV-C5 may affect the outcomes of preclinical and clinical trials. Indeed, transitioning from preclinical to clinical phases requires profound proof-of-concept studies in suitable animal models or patient cohorts. While several studies have shown that pre-existing antibodies against HAdV-C5 are common in the human population, ranging in prevalence from 61.3% to 94.0% depending on the geographic location [41,42,43], data from animals frequently used in preclinical studies are scarce.

While rhesus macaques are model organisms for preclinical studies, standardized screening for pre-existing neutralizing antibodies against HAdV-C5 is usually not performed, likely due to the absence of a suitable screening system. Compared to existing HAdV-C5 neutralization assays [26,27,28], we developed a high-throughput system based on an HAdV-C5 reporter vector expressing two reporter proteins. The expression of the fluorescent protein moxGFP allows for a quantitative readout of the number of transduced target cells via fluorescence microscopy and flow cytometry. In contrast, the expression of the luciferase NanoLuc offers a highly sensitive and quantitative readout at multiple time points, allowing for high-throughput screenings and longitudinal analysis. NanoLuc also affords high dynamic ranges and early readouts within 24 h post-transduction. We used this screening to analyze the prevalence of HAdV-C5-neutralizing antibodies in the sera of rhesus macaques from different primate centers. Interestingly, this prevalence significantly varied between the individual centers, indicating a difference in exposure frequency to HAdV-C5 or animal-specific adenovirus infections that induce cross-reactive antibodies. Our assay can also be used to measure vaccination-elicited anti-HAdV-C5 immunity in preclinical studies.

We also confirmed HAdV-C5 neutralization in the sera of rabbits, horses, cats, and dogs, showing the broad importance of prescreening for neutralization in multiple species. Indeed, previous research into both gene therapy and vaccinology has utilized all these species [29,44,45]. Future biomedical research in these species should be preceded by screening potential animals for HAdV-C5-neutralizing antibody responses.

Taken together, our data show that there is a critical need to perform prescreening for the prevalence of HAdV-C5 neutralizing antibodies, especially in preclinical in-vivo validation experiments for HAdV-C5-based viral vectors, and showcase the significant utility of our high-throughput HAdV-C5 neutralization assay for both preclinical and clinical research.

## Data Availability

The data presented in this study are available in the Supplementary Primary Data file. The sequence of the HAdV-C5-based reporter vector is available in the Supplementary Materials.

## Supplementary Materials

The following supporting information can be downloaded at: https://www.mdpi.com/article/10.3390/vaccines12020155/s1: Figure S1. Schematic of high-throughput HAdV-C5 neutralization assay. A schematic showing the assay with the recommended concentrations, volumes, and time intervals. Created with BioRender.; Figure S2. Viability of HepG2 cells transduced with different MOIs of the HAdV-C5-based reporter vector. HepG2 cells were transduced with the HAdV-C5-based reporter vector at different MOIs. After 72 h, the NanoLuc luciferase expression was quantified and the Cell-titer Blue viability assay performed; Figure S3. Viability of HepG2 cells incubated with the positive control serum and HAdV-C5-based reporter. The HAdV-C5 neutralization assay was performed as described in the Material and Methods section with the positive control serum (N = 9). Cell viability was measured following 72 h using a Cell-titer Blue viability assay; Figure S4. Determination of the ID50 from an ONPRC serum sample with HAdV-C5 neutralization. HepG2 cells were transduced with HAdV-C5-based reporter that had been incubated with varying dilutions of serum from rhesus macaque O1. Following 72 h, the NanoLuc expression was analyzed in triplicate. In addition, ID50 was calculated; Annotated GenBank sequence file of HAdV-C5-based reporter virus.
