# Supplementary figures and images for: High-Throughput Screening for the Prevalence of Neutralizing Antibodies against Human Adenovirus Serotype 5
Source: Vaccines (Basel). 2024 Feb 1;12(2):155. doi: 10.3390/vaccines12020155 (PMC10891882; doi:10.3390/vaccines12020155)

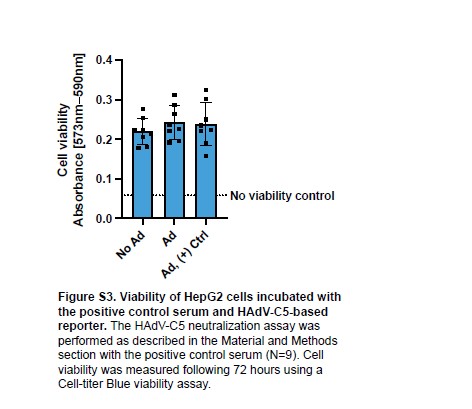

Supplement: Supplementary file 1 [file vaccines-12-00155-s001.zip › Figure S3.jpg]

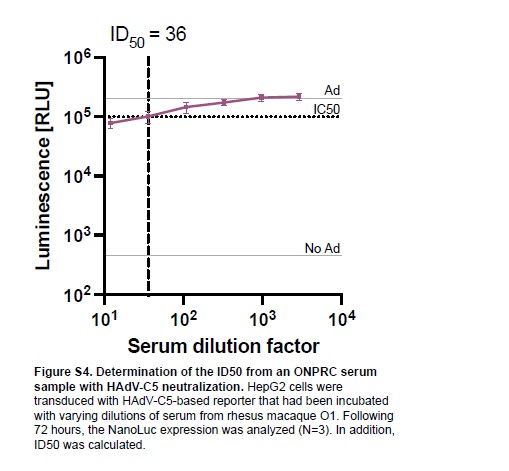

Supplement: Supplementary file 1 [file vaccines-12-00155-s001.zip › Figure S4.jpg]

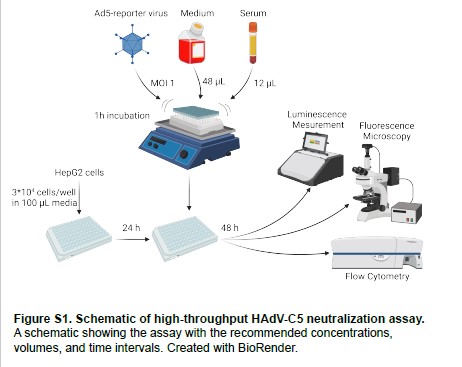

Supplement: Supplementary file 1 [file vaccines-12-00155-s001.zip › Figure S1.jpg]

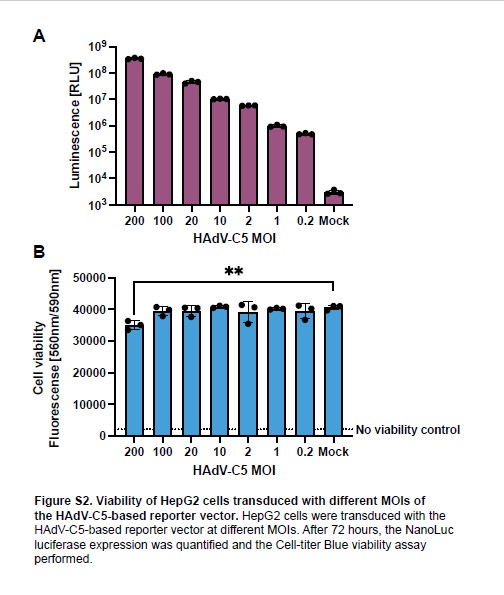

Supplement: Supplementary file 1 [file vaccines-12-00155-s001.zip › Figure S2.jpg]
